# A Cross Sectional Analysis of the Role of the Antimicrobial Peptide Cathelicidin in Lung Function Impairment within the ALIVE Cohort

**DOI:** 10.1371/journal.pone.0095099

**Published:** 2014-04-17

**Authors:** Allison A. Lambert, Gregory D. Kirk, Jacquie Astemborski, Enid R. Neptune, Shruti H. Mehta, Robert A. Wise, M. Bradley Drummond

**Affiliations:** 1 Department of Medicine, Division of Pulmonary and Critical Care, Johns Hopkins University, Baltimore, Maryland, United States of America; 2 Department of Medicine, Division of Infectious Diseases, Johns Hopkins University, Baltimore, Maryland, United States of America; 3 Department of Epidemiology, Johns Hopkins University, Baltimore, Maryland, United States of America; Leiden University Medical Center, Netherlands

## Abstract

**Background:**

Vitamin D deficiency is associated with reduced lung function. Cathelicidin, an antimicrobial peptide regulated by vitamin D, plays a role within the innate immune system. The association of cathelicidin with lung function decrement and respiratory infection is undefined.

We determined the independent relationship of cathelicidin with lung function.

**Methods:**

In a cross-sectional analysis of 650 participants in an urban observational cohort with high smoking prevalence, plasma 25(OH)-vitamin D and cathelicidin levels were measured from stored samples obtained within 6 months of spirometry study visits. Multivariable linear regression was used to determine the independent association between low cathelicidin (defined as the lowest quartile of the cohort) and absolute forced expiratory volume in 1 second (FEV1).

**Results:**

The mean age of the cohort was 49 years; 91% were black, 35% female and 41% HIV-infected. Participants with low cathelicidin had a 183 mL lower FEV1 compared to higher cathelicidin (p = 0.009); this relationship was maintained (115 ml lower; p = 0.035) after adjusting for demographics, BMI, and smoking. Neither HIV serostatus, heavy smoking history, nor 25(OH)-vitamin D levels were associated with cathelicidin levels. Participants with low cathelicidin had a greater prevalence of prior bacterial pneumonia (21% versus 14%; p = 0.047). Inclusion of pneumonia in adjusted models did not substantially reduce the FEV1 decrement observed with low cathelicidin (104 mL lower FEV1; p = 0.05). Lung function decrements associated with low cathelicidin were greatest among individuals with lower 25(OH)-vitamin D levels.

**Conclusions:**

In a cohort at risk for airflow obstruction, low cathelicidin was independently associated with lower FEV1. These clinical data support a mechanistic link between 25(OH)-vitamin D deficiency and lung function impairment, independent of pneumonia risk.

## Introduction

Obstructive lung diseases (OLDs), including asthma and chronic obstructive pulmonary disease, are prevalent conditions both nationally and globally [1]–[3]. Forced expiratory volume in 1 second (FEV1) provides a quantifiable measurement of disease severity and is the target of most therapies. Progressive FEV1 decline is associated with diminished quality of life and increased mortality [4], [5]. Even among persons without OLD, pulmonary infections may pose an independent risk for progressive lung function decline [6]. Understanding the independent risk factors for lung function impairment is necessary to mitigate the development and progression of chronic lung disease.

Low levels of vitamin D have been associated with an increased frequency of respiratory infection [7] and with reduced lung function [8]. These effects may be mediated through vitamin D regulation and activation of the innate immune system [9]. Cathelicidin is an antimicrobial peptide whose production and activation are dependent upon vitamin D [10], [11]. Secreted by neutrophils, macrophages and epithelial cells, cathelicidin regulates the innate immune system both through bactericidal, antiviral, anti-endotoxic and chemoattractant activities [12]–[14]. These activities may play a role in mitigating risk of respiratory infections and subsequent lung function decline. However, among persons with chronic lung disease or at risk for chronic lung disease, the association between cathelicidin and lung function impairment remains unclear.

In order to assess this relationship, we studied a well-characterized cohort at high risk for lung function impairment, vitamin D deficiency and respiratory infection. The AIDS Linked to the IntraVenous Experience (ALIVE) cohort is comprised of current or former injection drug users (IDUs) in Baltimore, Maryland with and without HIV infection who are closely followed with detailed behavioral and clinical data, stored blood samples, and spirometric measures of lung function [15]. This cohort has prevalent tobacco use and is at high risk for development of lung function impairment [16], [17]. Through analysis of this cohort, we determined the relationship between cathelicidin and lung function while accounting for potential confounders including prior pulmonary infections. We hypothesized that low cathelicidin would be independently associated with reduced lung function. Further, we examined for possible effect modification of the cathelicidin-lung function relationship by vitamin D level.

## Methods

### Study Population

Details of the ALIVE study have been reported previously [15]. Briefly, ALIVE is a prospective, observational cohort that has followed adult IDUs in Baltimore, MD since 1988. Since 2007, pre-bronchodilator spirometry has been performed at each ALIVE study visit.

This cross-sectional study measured cathelicidin levels from stored blood of 650 of the 915 ALIVE participants with prior spirometry and 25(OH)-vitamin D assay [18]. For efficiency of testing, all participants with HIV, OLD (as defined below) or both were selected for cathelicidin testing (n = 370). From the remaining disease free participants (n = 545), a random sample was selected for cathelicidin assay (n = 280). Participants included in this study had spirometry performed between January 2007 and December 2010 and blood samples were collected within 6.5 months of spirometry. This study was approved by the IRB of Johns Hopkins Bloomberg School of Public Health. All participants provided written informed consent.

### Measurements

Plasma cathelicidin levels were analyzed using a commercially available ELISA (Hycult Biotech, Uden, Netherlands); 25(OH)-vitamin D levels were measured using a radioimmunoassay (Diasorin, Stillwater, Minnesota, USA). Both assays were performed at Tufts Medical Center Core Laboratory. Pre-bronchodilator spirometry measurements, calculations and interpretation were consistent with ATS guidelines, as previously described [19]. Obstructive lung disease (OLD) was defined as pre-bronchodilator FEV1/FVC ratio less than 0.70 [20]. Demographic, clinical and laboratory data were collected at the time of spirometry measurement. Smoking patterns, IDU status and antiretroviral use in the prior 6 months were obtained through self-report. Self-report of respiratory infection was confirmed through medical record abstraction. Standardized medical record review classified infections as bacterial, *Pneumocystis* or other (e.g., viral, *Tuberculosis* or multifactorial). HIV serology testing (for HIV negatives) and CD4 count and HIV RNA testing were routinely performed at each study visit. HCV serology testing was performed within one year of 25(OH)-vitamin D testing or at study entry for more recent recruits.

### Statistical Analyses

The exposure of interest for this analysis was cathelicidin level and the primary outcome of interest was absolute FEV1 (mL). Absolute FEV1 was chosen, rather than FEV1% predicted, to allow comparison of the magnitude of the association between cathelicidin and FEV1 with the magnitude of the association between other established factors (age, race, sex) and FEV1. Cathelicidin levels were analyzed continuously and categorically with low cathelicidin defined as a value in the lowest quartile of the cohort. Results are presented as frequencies, mean (standard deviation) for normally distributed data and median (interquartile range [IQR]) for non-normally distributed data. Clinical and demographic characteristics were compared using the Student *t* test, Wilcoxon rank-sum tests, Mann-Whitney test, or Pearson χ^2^ as appropriate. Univariable linear regression was used to examine the association between clinical characteristics and FEV1. In order to determine the independent association of low cathelicidin with absolute FEV1, a multivariable linear regression model was generated which included statistically significant and clinically relevant covariates identified by univariable analysis. In univariate analysis, both height and BMI were strongly associated with absolute FEV1. BMI, rather than height, was included in models because BMI both adjusts for height and takes into account the potential impact of obesity on reduction in FEV1. Additional multivariable models separately included prior bacterial pneumonia and HIV to the base model, as well as both variables combined, to determine the impact of these covariates on the cathelicidin-lung function association.

In further analysis, we considered 25(OH)-vitamin D levels as a potential moderator variable; that is, a variable that influences the strength of the relationship between cathelicidin and lung function. 25(OH)-vitamin D levels were also modeled both continuously and categorically; 25(OH)-vitamin D deficiency was defined as a level <20 ng/mL [21]. Effect modification of the association between cathelicidin and lung function was examined by categorical 25(OH)-vitamin D levels with cut-offs of <10, 10–19 and >20 ng/mL [21]. We also evaluated pneumonia and HIV status as potential mediator variables. A two-sided p-value ≤0.05 was used to define statistical significance. All statistical analyses were performed using Stata version 12.0 (StataCorp, College Station, TX) and SAS version 9.0 (Cary, NC).

## Results

### Participant Characteristics

The mean age of the 650 ALIVE participants included in analysis was 49 years; 91% were black, 35% female and 41% HIV-infected (**Table 1**). The mean BMI was 26.4 kg/m^2^. Although 85.5% were current smokers, the mean FEV1 of the cohort was 91% predicted; 22% of the cohort met criteria for OLD (FEV1/FVC≤0.70). Among the HIV-infected participants, the median CD4+ cell count was 311 cells/mm^3^ (IQR: 177–502 cells/mm^3^) and the median viral load was 578 copies/mL (interquartile range [IQR]: <40 to 21950 copies/mL) with 102 participants (38%) having an undetectable viral load. For a complete description of the clinical and demographic characteristics stratified by HIV and OLD status see **Tables S1, S2 and S3 in File S1**. Compared to the 354 ALIVE participants not included in this analysis, the study was of similar age, gender, and race with the same frequency of ever smoking and recent injection drug use (data not shown). Participants included in the analysis were more likely to be taking a multivitamin than those not included (31% vs. 23%; p-value <0.01). Reflecting our selection criteria for testing, included participants were more likely to be HIV-infected (p-value <0.0001) and to have OLD (p-value <0.0001).

**Table 1:** Clinical and Demographic Characteristics of Study Participants.

Number of participants	650	
Age, years	48.6	(8.0)
Female, n (%)	227	(35)
Black race, n (%)	592	(91)
BMI, kg/m^2^	26.4	(6.2)
Smoking Status, n (%)*		
Current	556	(85.5)
Former	60	(9.2)
Never	34	(5.2)
Smoking, pack years	23.78	(16.9)
FEV1		
Absolute, L	2.73	(0.8)
% Predicted	90.1	(19)
FVC		
Absolute, L	3.59	(1.0)
% Predicted	95.5	(17)
Obstructive Lung Disease, n (%)	140	(22)
Current IDU, n (%)*	246	(38)
Hepatitis C antibody seropositive, n (%)	561	(86)
HIV-infected, n (%)	269	(41)
CD4+ cell count, cells/mm^3^ †	311	(177–502)
HIV-1 RNA level, copies/Ml †	578	(40.0–21950.0)
Undetectable Viral Load, n (%) †	102	(38)
HAART use, n (%) * †	148	(55)
Vitamin D		
Absolute Level, ng/mL	13.7	(9.04–20.3)
Deficiency (<20 ng/mL), n (%)	479	(74)
Cathelicidin level, ng/mL	36.0	(28.8–45.9)

Values presented as mean (SD) or median (IQR) unless indicated otherwise.

*In previous 6 months.

† Among participants with HIV.

Abbreviations: BMI, Body Mass Index; FEV1, Forced Expiratory Volume in 1 second; FVC, Forced Vital Capacity; HAART, Highly Active Antiretroviral Therapy; HIV, Human Immunodeficiency Virus; IDU, Injection Drug Use; IQR, interquartile range; SD, standard deviation; L, liters; RNA, ribonucleic acid.

The median cathelicidin level for the cohort was 36.0 ng/mL (IQR: 28.8–45.9 ng/mL) with a range of 4.35–1267 ng/mL. The median 25(OH)-vitamin D level was 13.7 ng/mL (IQR: 9.0–20.3 ng/mL) with a range from undetectable to 447.0 ng/mL. 74% of the cohort was 25(OH)-vitamin D deficient (defined as <20 ng/mL). 25(OH)-vitamin D and cathelicidin levels were weakly positively correlated (Spearman correlation coefficient 0.144; p-value <0.001) within the entire cohort. This correlation was slightly strengthened among those with 25(OH)-vitamin D levels ≥20 (0.163; p = 0.03) and attenuated among 25(OH)-vitamin D deficient participants (0.079; p = 0.08). Median cathelicidin levels were lower among participants with 25(OH)-vitamin D deficiency compared with those with adequate 25(OH)-vitamin D levels (35.2 ng/mL vs. 39.5 ng/mL; p = 0.003). Among those with 25(OH)-vitamin D deficiency, 79% had low cathelicidin levels (lowest quartile) compared to 72% of those with normal 25(OH)-vitamin D levels (p = 0.09).

### Univariable Correlates of Reduced FEV1

In univariable analysis, older age, black race, female gender, higher BMI and low cathelicidin levels were associated with lower absolute FEV1 (**Table 2**). Neither HIV serostatus, heavy smoking history (≥40pack-years), nor 25(OH)-vitamin D levels were associated with cathelicidin levels. For each 5-year increase in age, FEV1 decreased by 132 mL (95% CI: −168, −95; p <0.001). Black race was associated with a 700 mL lower FEV1 compared to non-black race (95% CI: −904, −496; p<0.001). Female gender was associated with an 838 mL lower FEV1 compared to male gender (95% CI: −946, −729; p<0.001). Every 1 kg/m^2^ increase in BMI was associated with a 17.7 mL decrease in FEV1 (95% CI: −27.4, −8.0; p-value <0.001). Examined continuously, a 100 ng/mL decrease in cathelicidin levels resulted in a 75 mL decrease in FEV1 (95% CI: 20.89, 129.18; p = 0.007). Participants with cathelicidin levels in the lowest quartile had a 183 mL lower FEV1 compared to those with higher cathelicidin levels (95% CI: −319, −46.4; p = 0.009).

**Table 2:** Association between Cohort Characteristics and Absolute FEV1 (mL).

	Unadjusted FEV1 Difference			Adjusted* FEV1 Difference		
Predictor	(95% CI)		p-value	(95% CI)		p-value
Age, per 5 years older	−132	(−168, −95)	<0.0001	−162	(−194, −130)	<0.0001
Black	−700	(−904, −496)	<0.0001	−335	(−510, −160)	<0.001
Female	−838	(−946, −729)	<0.0001	−911	(−1013, −810)	<0.0001
BMI, per kg/m^2^	−17.7	(−27, −8)	<0.001	−6.01	(−14, 2)	0.128
Low Cathelicidin Level†	−183	(−319, −46)	0.009	−115	(−221, −8)	0.035

*Model adjusted for other predictors in the table.

† Defined as lowest quartile compared with remaining population.

Abbreviations: BMI, Body Mass Index; CI, Confidence Interval; FEV1, Forced Expiratory Volume in 1 second.

When comparing individuals who met spirometric criteria for OLD to those who did not, there was no difference in median cathelicidin level when stratifying by OLD status. We observed no association between HIV serostatus and median cathelicidin level (35.5 ng/mL for HIV-infected vs. 36.4 for HIV-uninfected; p = 0.12) or with the prevalence of low cathelicidin among those with HIV compared to those without HIV (29% vs. 24%; p = 0.45).

### Multivariable Model Examining Independent Cathelicidin-FEV1 Relationship

After adjusting for relevant covariates (age, race, gender, and BMI), participants with cathelicidin levels in the lowest quartile had a 115 mL lower FEV1 compared to those with higher cathelicidin levels (95% CI: −221, −7.91; p = 0.035; **Table 2**). Aside from BMI, which lost statistical significance, all other covariates related to FEV1 in univariable analysis maintained statistically significant association with FEV1 in multivariable models. For each 5-year increase in age, FEV1 decreased by 162 mL (95% CI: −194, −130; p-value <0.001). Black race was associated with a 335 mL lower FEV1 (95% CI: −510, −160; p-value <0.001). Female gender was associated with a 911 mL lower FEV1 (95% CI: −1013, −810; p-value <0.001). Addition of heavy smoking history (≥40 pack-years) did not attenuate the FEV1 decrement observed with low cathelicidin levels (106 mL lower).

### Effect Modification by 25(OH)-vitamin D Levels

**Figure 1:**
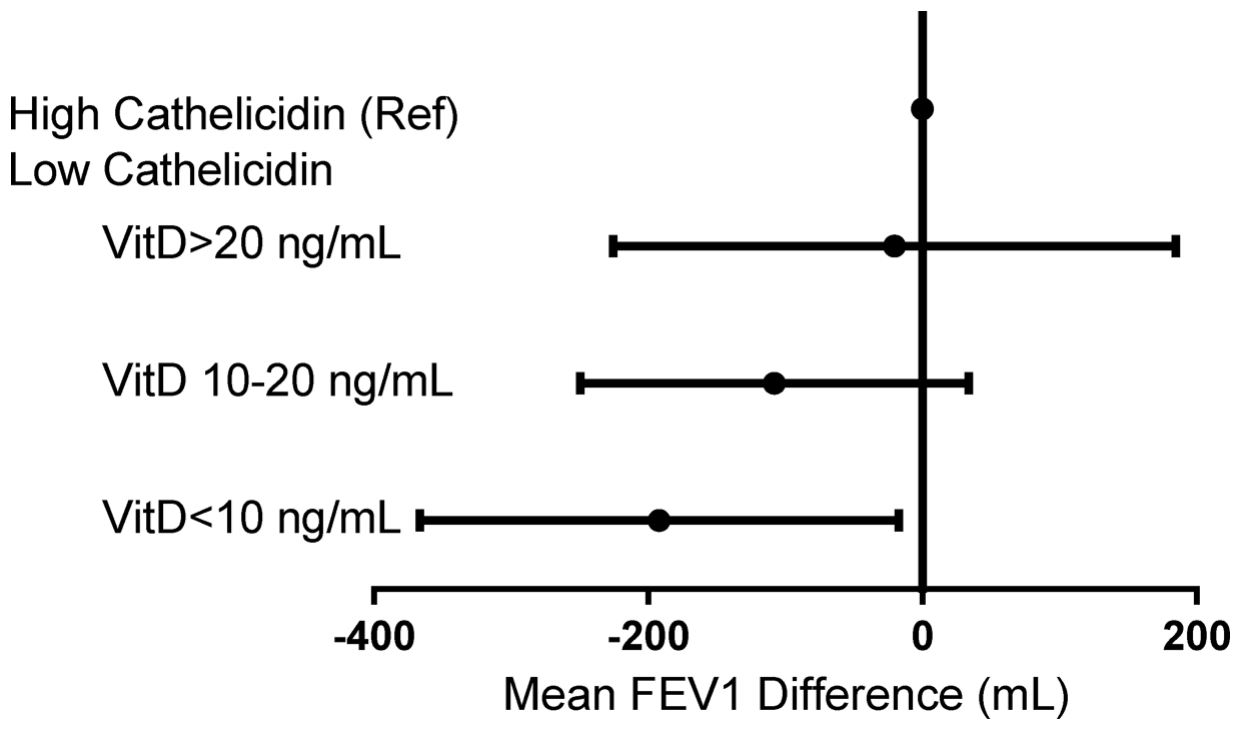
Adjusted association between cathelicidin/vitamin D categories and absolute FEV1. When categorizing participants with low cathelicidin levels by vitamin D level and comparing to participants with high cathelicidin level, a dose-response reduction in FEV1 is observed. Values adjusted for age, race, gender and body mass index. Width of line represents 95% CI. (Ref =  reference; VitD =  vitamin D; FEV1 =  forced expiratory volume in one second).

25(OH)-vitamin D, when examined either continuously or categorically as deficient versus sufficient, was not associated with a difference in FEV1. There also was no difference in the prevalence of participants with 25(OH)-vitamin D deficiency when comparing those with OLD to those without OLD (29% vs. 25%; p = 0.87). However, 25(OH)-vitamin D deficiency was increased among HIV-uninfected participants compared with HIV-infected participants (78% vs. 67%; p = 0.002). When subgrouping participants with low cathelicidin levels into categories of 25(OH)-vitamin D deficiency and comparing to participants with high cathelicidin levels, a dose-response reduction in FEV1 was observed (**Figure 1**). When categorizing participants with low cathelicidin levels by 25(OH)-vitamin D level and compared to participants with high cathelicidin level, a dose-response reduction in FEV1 was observed (Figure 1). Among participants with low cathelicidin levels, those with the 25(OH)-vitamin D<10 ng/mL demonstrated the most substantial FEV1 decrement (192 ml; 95% CI −366 ml to −17 ml; p = 0.03). Participants with moderate 25(OH)-vitamin D deficiency (10–19 ng/ml) had 108 ml lower FEV1 (95% CI −250 to 34 ml; p = 0.14) while low cathelicidin-25(OH)-vitamin D sufficient participants had a 20 ml lower FEV1 (95% CI −225 to 185 ml; p = 0.84). Addition of season of 25(OH)-vitamin D measurement to all univariable and multivariable models did not change effect estimates or statistical significance (data not shown).

### Moderation of Cathelicidin Effect by Pneumonia and HIV Status

Because lower cathelicidin levels may increase the risk for bacterial pneumonia and because pneumonia is well-recognized to negatively impact lung function, we evaluated whether prior pneumonia mediated some of the association between cathelicidin and FEV1. Bacterial pneumonia comprised 87% of confirmed respiratory infections. Of the103 cases of lower respiratory tract infections we observed, only 5 occurred within 30 days and an additional 2 within 45 days of cathelicidin measurement. Participants with low cathelicidin had a greater prevalence of prior pneumonia (20% versus 14%; p = 0.047). As well, prior bacterial pneumonia was associated with a 424 mL lower FEV1 (95% CI: −587.3, −261.7; p-value <0.001) in univariable analysis and a 248 mL lower FEV1 (95% CI: −377.5, −119.1; p-value <0.001) in a multivariable model including age, race, gender, and BMI. Inclusion of pneumonia in the multivariable model slightly attenuated the magnitude of the association between low cathelicidin and lower FEV1 (104 mL decrease in FEV1; 95% CI: −209.6, 0.01; p = 0.05). Inclusion of HIV serostatus in the multivariable model slightly attenuated the strength of the effect but did not moderate the statistical significance of the observed association between low cathelicidin and lower FEV1 (111 mL decrease in FEV1; 95% CI: 218.2, 4.74; p <0.0001). In a model including HIV and pneumonia, as well as age, race, sex and BMI from our base multivariable model, low cathelicidin was associated with a 103 mL lower FEV1 (95% CI: −208.7, 3.01; p = 0.057; **Table 3**). Multivariable models including pneumonia and HIV serostatus individually with the base multivariable model are available in **Table S4 in File S1**.

**Table 3:** Association between Cohort Characteristics, Pneumonia, HIV and Absolute FEV1 (mL).

	Unadjusted FEV1 Difference			Adjusted* FEV1 Difference		
Predictor	(95% CI)		p-value	(95% CI)		p-value
Age, per 5 years older	−132	(−168, −95)	<0.0001	−159	(−191, −127)	<0.0001
Black	−700	(−905, −496)	<0.0001	−319	(−494, −144)	<0.001
Female	−838	(−946, −729)	<0.0001	−886	(−987, −784)	<0.0001
BMI, per kg/m^2^	−17.7	(−27, −8)	<0.001	−7.86	(−16, −0.1)	0.047
Low Cathelicidin Level†	−183	(−320, −46)	0.009	−103	(−209, 3)	0.057
Prior Pneumonia‡	−424	(−588, −261)	<0.0001	−240	(−373, −108)	<0.001
HIV infection	−73.9	(−196, 48)	0.24	−27.8	(−125, 69)	0.57

*Model adjusted for other predictors in the table.

† Defined as lowest quartile compared with remaining population.

‡ Occurring anytime in the past.

Abbreviations: BMI, Body Mass Index; CI, Confidence Interval; FEV1, Forced Expiratory Volume in 1 second; HIV, Human Immunodeficiency Virus.

### Effect of Multivitamin Supplementation on 25(OH)-vitamin D and Cathelicidin Levels

Given the hypothesized regulatory role of 25(OH)-vitamin D upon cathelicidin, we further examined the relationship between these two biomarkers and multivitamin use. Multivitamin use was associated with a higher median cathelicidin level (38.0 ng/mL vs. 35.2 ng/mL; p = 0.03). Multivitamin use was also more prevalent among participants with normal 25(OH)-vitamin D levels compared to those who were 25(OH)-vitamin D deficient (42% vs. 27%; p-value <0.001). Multivitamin use was not independently associated with a change in FEV1 (p = 0.96) when added to the base model including age, race, sex and BMI.

## Discussion

In this study of 650 individuals with plasma cathelicidin, 25(OH)-vitamin D and spirometry measurements, we observed that low cathelicidin levels were independently associated with reduced FEV1. We observed no association between cathelicidin levels and HIV serostatus. While low cathelicidin levels were associated with a history of bacterial pneumonia, inclusion of pneumonia in adjusted models did not substantially reduce the FEV1 decrement seen with low cathelicidin. Among individuals with low cathelicidin levels, lower 25(OH)-vitamin D levels were associated with lower FEV1. Finally, multivitamin use was associated with higher cathelicidin levels.

To our knowledge, this is the first large study to examine the relationship between plasma cathelicidin level and lung function decrement. We observed a substantial reduction in FEV1 among participants with low cathelicidin levels, which was maintained after adjusting for age, race, gender, HIV serostatus and BMI. Prior studies have focused on induced sputum or bronchoalveolar lavage cathelicidin and its association with FEV1 [22]–[24]. In these studies, the primary endpoint was the presence of OLD, not absolute FEV1. These authors found higher levels of cathelicidin to be associated with established OLD. We did not observe an association between cathelicidin levels and OLD. This may reflect that the association between cathelicidin and lung function is relevant prior to development of overt lung disease, and other factors may impact FEV1 to a greater degree once OLD is present. There may be several other explanations for our differing observations. First, few data exist regarding the relationship between plasma and sputum cathelicidin [25], [26]. Second, prior studies have focused on patient populations with specific exposures, such as farmers, patients with cystic fibrosis, or smokers in acute care facilities [22], [23]. The participants in this study are predominantly African American, tobacco dependent and 25(OH)-vitamin D deficient. Lastly, these studies examined fewer individuals than our study.

We observed an association between low cathelicidin levels and history of bacterial pneumonia. The antimicrobial role of cathelicidin is well-established [12], [27], [28]. Cathelicidin has activity against a broad range of gram positive and gram negative bacteria, fungi, and even viruses [28]–[30]. Its activity against *Mycobacterium tuberculosis* and *Pseudomonas* [31], [32] is often highlighted because these infections are associated with chronic lung disease and progressive lung function decline [33]. In the setting of OLD exacerbation caused by non-typeable *Haemophilus influenza* and *Moraxella catarrrhalis*, sputum cathelicidin levels are increased [26]. Serum cathelicidin has been shown to recruit polymorphonuclear leukocytes, monocytes, mast cells, and CD4+ T-lymphocytes; these chemotactic properties have implicated its role in chronic inflammation [34], [35]. Importantly, in our analysis, low cathelicidin is associated with decreased FEV1, even after accounting for prior bacterial pneumonia. While this relationship did not maintain statistical significance in likely underpowered secondary analyses, the magnitude of the association suggests that cathelicidin deficiency may directly lead to FEV1 reduction, independent of increased risk of respiratory infections. We did not observe an association between HIV serostatus and cathelicidin levels which suggests that HIV infection may not play a key role in cathelicidin modulation, though the high prevalence of 25(OH)-vitamin D deficiency and heterogeneous HIV disease severity limits rigorous exploration of this association. Other studies have highlighted the impact of HIV infection on cathelicidin level and function in other body systems [36], [37].

Controversy exists in the literature regarding the role of vitamin D in the pathogenesis of chronic lung disease. Several cross-sectional studies have shown a relationship between vitamin D deficiency and reduced lung function [8], [38]–[40]. Longitudinal data has shown that vitamin D deficiency significantly modified the effect of smoking on lung function decline; however an independent association between vitamin D deficient and FEV1 was absent [41]. In the same study, among current smokers, vitamin D deficiency was associated with significantly lower FEV1 compared to smokers with sufficient vitamin D levels, suggesting that replete vitamin D levels may protect against lung damage from smoking. In a prospective cohort study of patients with non-cystic fibrosis bronchiectasis, vitamin D deficiency was associated with increased frequency of chronic bacterial colonization, greater quantitative bacterial load, more frequent outpatient exacerbations, and elevated markers of airway inflammation [7]. We did not observe an association between 25(OH)-vitamin D deficiency and FEV1, likely due to the high prevalence of 25(OH)-vitamin D deficiency within our study population. This high prevalence of 25(OH)-vitamin D deficiency may have also weakened the positive statistically significant correlation we observed between 25(OH)-vitamin D levels and cathelicidin levels due to the limited range of 25(OH)-vitamin D levels. There may be a threshold of vitamin D level whereby the correlation of vitamin D and cathelicidin is strong above a certain level and weaker below such level (or *vice versa*). However, among those with low cathelicidin, we did observe a clear dose-response relationship between lower 25(OH)-vitamin D levels and reduced lung function. While compelling evidence suggests that vitamin D replacement may improve lung outcomes, our findings highlight that there may be a subset of individuals who will achieve maximal benefit. We hypothesize that the combination of low 25(OH)-vitamin D and low cathelicidin may identify those at risk for lung function impairments and who may benefit from vitamin D replacement.

**Figure 2:**
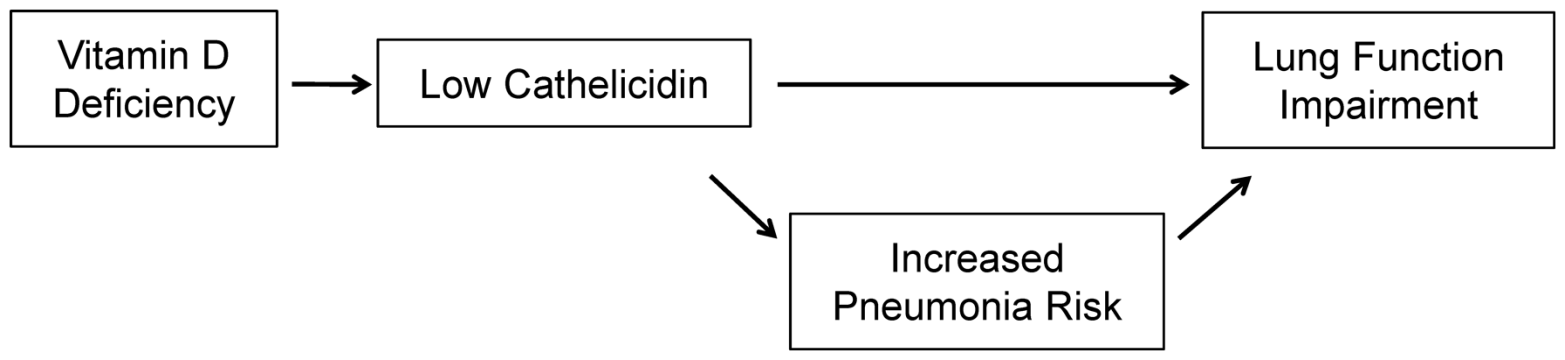
Conceptual framework for the association between cathelicidin, vitamin D, pneumonia, and lung function.

The role of cathelicidin in the vitamin D deficiency-FEV1 relationship is not well established, though studies have begun to characterize these associations [7], [22], [23], [42]. In our study, 25(OH)-vitamin D deficiency was associated with lower cathelicidin levels. Low cathelicidin levels were associated with prior bacterial pneumonia and independently associated with reduced FEV1. These findings are consistent with the conceptual pathway of disease (illustrated in **Figure 2**) in which 25(OH)-vitamin D deficiency leads to reduced cathelicidin expression and function, which induces lung function decrements through recurrent infection and inflammation. Given the complex downstream effectors of vitamin D, it is possible that there are cathelicidin-independent vitamin D effects impacting lung function. Non-infectious pathways for low cathelicidin-associated lung function impairment remain unclear and under-explored.

We observed that individuals reporting multivitamin use had higher cathelicidin levels, suggesting that vitamin D replacement may successfully increase cathelicidin levels. Other studies have shown that repletion of vitamin D upregulates cathelicidin expression in human tracheobronchial epithelial cells in vitro, as well as in the skin of atopic dermatitis patients [42], [43]. Our findings reinforce the importance of understanding the impact of nutritional interventions on overall lung health.

This study has limitations. The cross-sectional design prevents inferences regarding causality or longitudinal effects of cathelicidin on lung function decline. The distinct nature of the ALIVE cohort – an urban population that is largely African American, tobacco dependent, and 25(OH)-vitamin D deficient with a former or active injection drug use history – may limit the generalizability of our results to other populations at risk for chronic lung disease. However, ALIVE participants represent an underserved, understudied population that is at risk for chronic lung disease, 25(OH)-vitamin D deficiency and respiratory infections [17]. While the homogeneity within the cohort limits inferences regarding race and smoking associations, this same homogeneity decreases the likelihood of false associations related to unmeasured confounders. Similarly, the high prevalence of 25(OH)-vitamin D deficient participants limits the ability to rigorously define the potential interaction between 25(OH)-vitamin D and the cathelicidin-lung function relationship. Pre- and post-bronchodilator spirometry was not measured, limiting the distinction between reversible and fixed airflow obstruction. Therefore our findings may not be representative of post-bronchodilator spirometry measurements. Cathelicidin may be influenced by other inflammatory markers such as blood neutrophils which are not accounted for in our analysis. Our statistical model included BMI which may not fully adjust for the impact of height upon FEV1; however, this approach does more fully account for the impact of obesity on lung function impairment than height. Likely, local lung cathelicidin levels are more relevant than samples obtained peripherally. Our study did not have concurrent blood and bronchoalveolar cathelicidin measurements. Prior data reported an association between peripheral blood and BAL cathelicidin levels [25]. Multivitamin use was self-reported and is therefore subject to expectation and recall bias. Despite these limitations, the study presented here represents, to our knowledge, the largest analyses of the relationship between plasma cathelicidin and lung function.

In summary, we have observed that low cathelicidin levels are independently associated with lower FEV1. The severity of lung function impairment among those with low cathelicidin was worse with more substantial 25(OH)-vitamin D deficiency. The relationship between low cathelicidin and decreased FEV1 has distinct implications among a cohort of patients at high risk for both infection and chronic lung disease. The association of multivitamin intake and cathelicidin levels incites speculation about treatment opportunities. These findings contribute to the ongoing elucidation of the role of cathelicidin in innate immunity and highlight a potentially modifiable biomarker for increased risk of infection and lung function impairment.

## Supporting Information

File S1 **This file contains Table S1-Table S4.** Table S1, Clinical and Demographic Characteristics of Study Participants by Disease Status. Table S2, Clinical and Demographic Characteristics of Study Participants by HIV Serostatus. Table S3, Clinical and Demographic Characteristics of Study Participants by OLD Status. Table S4, Additional Models- Pneumonia and HIV Serostatus.
(DOCX)
