# Supplementary material for: A Cross Sectional Analysis of the Role of the Antimicrobial Peptide Cathelicidin in Lung Function Impairment within the ALIVE Cohort
Source: PLoS One. 2014 Apr 17;9(4):e95099. doi: 10.1371/journal.pone.0095099 (PMC3990590; doi:10.1371/journal.pone.0095099)
Supplement: File S1 — This file contains Table S1-Table S4. Table S1, Clinical and Demographic Characteristics of Study Participants by Disease Status. Table S2, Clinical and Demographic Characteristics of Study Participants by HIV Serostatus. Table S3, Clinical and Demographic Characteristics of Study Participants by OLD Status. Table S4, Additional Models- Pneumonia and HIV Serostatus. (DOCX) [file pone.0095099.s001.docx]

**A CROSS SECTIONAL ANALYSIS OF THE ROLE OF THE ANTIMICROBIAL PEPTIDE CATHELICIDIN IN LUNG FUNCTION IMPAIRMENT WITHIN THE ALIVE COHORT**

Allison A. Lambert^1^, Gregory D. Kirk^2,3^, Jacquie Astemborski^3^, Enid R. Neptune^1^, Shruti H. Mehta^3^, Robert A. Wise^1^, M. Bradley Drummond^1^

^1^Department of Medicine; Division of Pulmonary and Critical Care; Johns Hopkins University, Baltimore, MD, USA; ^2^Department of Medicine; Division of Infectious Diseases; Johns Hopkins University, Baltimore, MD, USA; ^3^Department of Epidemiology; Johns Hopkins University, Baltimore, MD, USA

**Corresponding Author:**

Allison Ann Lambert, MD

1830 East Monument St, 5^th^ Floor

Baltimore, MD 21205 Phone: 410-955-3467 Fax: 410-955-0036

Email: alamber5@jhmi.edu

| Table S1 Clinical and Demographic Characteristics of Study Participants by Disease Status | | | | | | | | | | |
| --- | --- | --- | --- | --- | --- | --- | --- | --- | --- | --- |
|  | | | OLD Absent | | OLD Present | | OLD Absent | | OLD Present | |
|  | | | HIV Uninfected | | HIV Uninfected | | HIV Infected | | HIV Infected | |
| Number of participants | | | 280 | | 101 | | 230 | | 39 | |
| Age, years | | | 48.4 | (8) | 51.2 | (9) | 47.6 | (6) | 49.8 | (9) |
| Female, n (%) | | | 96 | (34) | 31 | (31) | 90 | (39) | 10 | (26) |
| Black race, n (%) | | | 251 | (90) | 87 | (86) | 220 | (96) | 34 | (87) |
| BMI, kg/m^2^ | | | 27.5 | (6) | 25.1 | (5) | 25.8 | (6) | 25.1 | (6) |
| Smoking Status, n (%)* | | |  |  |  |  |  |  |  |  |
|  | Current | | 240 | (86) | 88 | (87) | 194 | (84) | 34 | (87) |
|  | Former | | 25 | (9) | 11 | (11) | 22 | (10) | 2 | (5) |
|  | Never | | 15 | (5) | 2 | (2) | 14 | (6) | 3 | (8) |
| Smoking, pack years | | | 24.0 | (17) | 27.0 | (18) | 22.3 | (16) | 22.9 | (16) |
| FEV1 | | |  |  |  |  |  |  |  |  |
|  | Absolute, L | | 2.92 | (0.7) | 2.29 | (0.8) | 2.79 | (0.7) | 2.04 | (0.7) |
|  | % Predicted | | 95.7 | (15) | 72.7 | (19) | 94.6 | (16) | 67.3 | (20) |
| FVC | | |  |  |  |  |  |  |  |  |
|  | Absolute, L | | 3.70 | (0.9) | 3.60 | (1.2) | 3.51 | (0.9) | 3.25 | (1.0) |
|  | % Predicted | | 97.5 | (16) | 91.8 | (19) | 96.0 | (17) | 87.5 | (20) |
| Current IDU, n (%)* | | | 110 | (39) | 44 | (44) | 81 | (35) | 11 | (28) |
| Hepatitis C antibody seropositive, n (%) | | | 220 | (79) | 86 | (85) | 218 | (95) | 37 | (95) |
| Vitamin D | | |  |  |  |  |  |  |  |  |
|  | Absolute Level, ng/mL | | 13.6 | (9.3-19.4) | 13.8 | (9.4-18.6) | 14.6 | (9.0-22.2) | 11.4 | (7.3-22.4) |
|  | Deficiency (<20 ng/mL), n (%) | | 217 | (78) | 81 | (80) | 154 | (67) | 27 | (69) |
| Cathelicidin | | |  |  |  |  |  |  |  |  |
|  | | Absolute Level, ng/mL | 36.3 | (29.3-46.7) | 37.0 | (28.8-47.8) | 35.9 | (28.6-45.6) | 33.3 | (24.6, 40.0) |
|  | | Low Level, n (%) ‡ | 66 | (24) | 26 | (26) | 63 | (27) | 14 | (36) |
| Values presented as mean (SD) or median (IQR) unless indicated otherwise.  * In previous 6 months.  † Among participants with HIV.  ‡ A cathelicidin level within the lowest quartile of the entire cohort of participants  Abbreviations: BMI, Body Mass Index; FEV1, Forced Expiratory Volume in 1 second; FVC, Forced Vital Capacity; HAART, Highly Active Antiretroviral Therapy; HIV, Human Immunodeficiency Virus; IDU, Injection Drug Use; IQR, interquartile range; SD, standard deviation; L, liters; RNA, ribonucleic acid | | | | | | | | | | |

| Table S2 Clinical and Demographic Characteristics of Study Participants by HIV Serostatus | | | | | |
| --- | --- | --- | --- | --- | --- |
|  | | HIV Uninfected | | HIV Infected | |
| Number of participants | | 381 | | 269 | |
| Age, years | | 49.1 | (9) | 47.9 | (7) |
| Female, n (%) | | 127 | (33) | 100 | (37) |
| Black race, n (%) | | 338 | (89) | 254 | (94) |
| BMI, kg/m^2^ | | 26.9 | (6) | 25.7 | (6) |
| Smoking Status, n (%)* | |  |  | | |
|  | Current | 328 | (86) | 228 | (85) |
|  | Former | 36 | (9) | 24 | (9) |
|  | Never | 17 | (4) | 17 | (6) |
| Smoking, pack years | | 24.8 | (18) | 22.4 | (16) |
| FEV1 | |  |  | | |
|  | Absolute, L | 2.76 | (0.8) | 2.68 | (0.7) |
|  | % Predicted | 89.6 | (19) | 90.7 | (19) |
| FVC | |  |  | | |
|  | Absolute, L | 3.68 | (1.0) | 3.47 | (0.9) |
|  | % Predicted | 96.0 | (17) | 94.8 | (17) |
| Obstructive Lung Disease, n (%) | | 101 | (27) | 39 | (15) |
| Current IDU, n (%)* | | 154 | (40) | 92 | (34) |
| Hepatitis C antibody seropositive, n (%) | | 306 | (80) | 255 | (95) |
| Vitamin D | |  |  | | |
|  | Absolute Level, ng/mL | 13.6 | (9.3-19.0) | 14.1 | (8.9-22.2) |
|  | Deficiency (<20 ng/mL), n (%) | 298 | (78) | 181 | (67) |
| Cathelicidin | |  |  |  |  |
|  | Absolute Level, ng/mL | 36.4 | (29.2-47.0) | 35.5 | (28.4-44.6) |
|  | Low Level, n (%) ‡ | 92 | (24) | 77 | (29) |
| Values presented as mean (SD) or median (IQR) unless indicated otherwise.  * In previous 6 months.  † Among participants with HIV.  ‡ A cathelicidin level within the lowest quartile of the entire cohort of participants  Abbreviations: BMI, Body Mass Index; FEV1, Forced Expiratory Volume in 1 second; FVC, Forced Vital Capacity; HAART, Highly Active Antiretroviral Therapy; HIV, Human Immunodeficiency Virus; IDU, Injection Drug Use; IQR, interquartile range; SD, standard deviation; L, liters; RNA, ribonucleic acid | | | | | |

| Table S3 Clinical and Demographic Characteristics of Study Participants by OLD Status | | | | | | |
| --- | --- | --- | --- | --- | --- | --- |
|  | | | OLD Absent | | OLD Present | |
| Number of participants | | | 510 | | 140 | |
| Age, years | | | 48.1 | (8) | 50.8 | (9) |
| Female, n (%) | | | 186 | (36) | 41 | (29) |
| Black race, n (%) | | | 471 | (92) | 121 | (86) |
| BMI, kg/m^2^ | | | 26.7 | (6) | 25.1 | (5) |
| Smoking Status, n (%)* | | |  |  | | |
|  | Current | | 434 | (85) | 122 | (87) |
|  | Former | | 47 | (9) | 13 | (9) |
|  | Never | | 29 | (6) | 5 | (4) |
| Smoking, pack years | | | 23.2 | (17) | 25.9 | (17) |
| FEV1 | | |  |  | | |
|  | Absolute, L | | 2.86 | (0.7) | 2.22 | (0.8) |
|  | % Predicted | | 95.2 | (16) | 71.2 | (19) |
| FVC | | |  |  | | |
|  | Absolute, L | | 3.62 | (0.9) | 3.50 | (1.1) |
|  | % Predicted | | 96.9 | (16) | 90.6 | (19) |
| Current IDU, n (%)* | | | 191 | (37) | 55 | (39) |
| Hepatitis C antibody seropositive, n (%) | | | 438 | (86) | 123 | (88) |
| HIV-infected, n (%) | | | 230 | (45) | 39 | (28) |
|  | CD4+ cell count, cells/mm^3^  † | | 311 | (177-496) | 305 | (173-530) |
|  | HIV-1 RNA level, copies/mL † | | 630 | (40-21800) | 422 | (40-36000) |
|  | Undetectable Viral Load, n (%) † | | 84 | (37) | 18 | (46) |
|  | HAART use, n (%) *† | | 127 | (55) | 21 | (54) |
| Vitamin D | | |  |  | | |
|  | Absolute Level, ng/mL | | 13.8 | (9.2-20.6) | 13.1 | (8.9-19.3) |
|  | Deficiency (<20 ng/mL), n (%) | | 371 | (73) | 108 | (77) |
| Cathelicidin | | |  |  |  |  |
|  | | Absolute Level, ng/mL | 36.0 | (29.0-46.0) | 35.8 | (27.5-45.1) |
|  | | Low Level, n (%) ‡ | 129 | (25) | 40 | (29) |
| Values presented as mean (SD) or median (IQR) unless indicated otherwise.  * In previous 6 months.  † Among participants with HIV.  ‡ A cathelicidin level within the lowest quartile of the entire cohort of participants  Abbreviations: BMI, Body Mass Index; FEV1, Forced Expiratory Volume in 1 second; FVC, Forced Vital Capacity; HAART, Highly Active Antiretroviral Therapy; HIV, Human Immunodeficiency Virus; IDU, Injection Drug Use; IQR, interquartile range; SD, standard deviation; L, liters; OLD, Obstructive Lung Disease; RNA, ribonucleic acid | | | | | | |

| Table S4 Additional Models- Pneumonia and HIV Serostatus | | | | | | | | | |
| --- | --- | --- | --- | --- | --- | --- | --- | --- | --- |
|  | BASE MODEL | | | BASE MODEL | | | BASE MODEL | | |
|  |  | | | WITH PNEUMONIA | | | WITH HIV SEROSTATUS | | |
| Predictor | Adjusted* FEV1 difference (95% CI) | | p-value | Adjusted* FEV1 difference (95% CI) | | p-value | Adjusted* FEV1 difference (95% CI) | | p-value |
| Age, per 5 years older | - 162 | (-194, -130) | <0.0001 | - 158 | (-189, -126) | <0.0001 | - 164 | (-196, -133) | <0.0001 |
| Black | - 335 | (-510, -160) | <0.001 | - 325 | (-499, -152) | <0.001 | - 320 | (-496, -143) | <0.001 |
| Female | - 911 | (-1013, -810) | <0.0001 | - 866 | (-988, -785) | <0.0001 | - 909 | (-1011, -807) | <0.0001 |
| BMI, per kg/m^2^ | - 6.01 | (-13.8, 1.73) | 0.128 | - 7.68 | (-15.4, 0.03) | 0.051 | - 6.56 | (-14.34, 1.21) | 0.098 |
| Low Cathelicidin Level† | - 115 | (-221, -7.91) | 0.035 | - 104 | (-210, 1.91) | 0.054 | - 111 | (-218, -4.74) | 0.041 |
| Prior Pneumonia‡ |  | | | - 248 | (-378, -119) | <0.001 |  | |  |
| HIV infection |  | | |  |  |  | - 65.25 | (-161, 30.48) | 0.181 |
| * Model adjusted for other predictors in the table.  † Defined as lowest quartile compared with remaining population  ‡ Occurring anytime in the past  Abbreviations: BMI, Body Mass Index; CI, Confidence Interval; FEV1, Forced Expiratory Volume in 1 second | | | | | | | | | |
